# Coronary artery calcification in CKD-5D patients is tied to adverse cardiac function and increased mortality

**DOI:** 10.5414/CN108940

**Published:** 2016-11-02

**Authors:** Paul Anaya, Gustav A. Blomquist, Daniel L. Davenport, Marie-Claude Monier-Faugere, Vincent L. Sorrell, Hartmut H. Malluche

**Affiliations:** 1 Division of Cardiovascular Medicine,; 2 Departments of Internal Medicine,; 3 Radiology, and; 4 Surgery, and; 5 Division of Nephrology, Bone and Mineral Metabolism, University of Kentucky, Lexington, KY, USA

**Keywords:** echocardiography, longitudinal strain, dialysis, CKD-5D, FGF-23

## Abstract

Background: Coronary artery calcification (CAC) is common in patients with chronic kidney disease on hemodialysis (CKD-5D) and is an important predictor of mortality. However, cardiac functional links between CAC and mortality have not been well established. This study tested the hypothesis that CAC increases mortality by adversely affecting cardiac function. Methods: Patients were recruited from 37 regional dialysis centers. 2-D and Doppler echocardiographic analyses were performed, and CAC was measured using 64-slice computed tomography. Relationships between CAC and echocardiographic measures of left ventricular (LV) function were analyzed. Survival was assessed with median follow-up of 37 months. Results: There were 157 patients: 59% male, 46% Caucasian, 48% diabetic. Median age was 55 years, and median duration of CKD-5D was 45 months. Agatston CAC scores > 100 were found in 69% of patients, with 51% having a score > 400. CAC was associated with measures of LV systolic and diastolic function (global longitudinal strain (GLS; rho = 0.270, p = 0.004)), peak LV systolic velocity (rho = –0.259, p = 0.004), and estimate of LV filling pressure (E:E’; rho = 0.286, p = 0.001). Multivariate regression confirmed these relationships after adjustment for age, gender, LV ejection fraction, and coronary artery disease. Valvular calcification varied linearly with CAC (p < 0.05). Both LV diastolic and systolic functional measures were significant predictors of mortality, the strongest of which was LV diastolic dysfunction. Conclusions: These findings show a link between CAC, cardiac function, and mortality in CKD-5D. LV diastolic function (E:E’), peak LV systolic velocity, and GLS are independent predictors of mortality. Valvular calcification may be an important marker of CAC in CKD-5D. These effects on cardiac function likely explain the high mortality with CKD-5D and describe a potentially-valuable role for echocardiography in the routine management of these patients.

## Short summary 

CKD-5D is characterized by a high coronary artery calcification (CAC) burden that is strongly associated with adverse global cardiac function. Additionally, cardiac valvular calcification detected by echocardiography is common in CKD-5D and is linked to both CAC severity and abnormal LV function. Abnormal cardiac function manifested from CKD-5D adversely impacts survivability. Therefore, these findings support the recommendation that a comprehensive echocardiographic evaluation be part of the cardiovascular risk stratification in CKD-5D patients. 

## Introduction 

Patients with chronic kidney disease (CKD) are at increased risk of developing cardiovascular (CV) disease and have a higher mortality attributed to cardiac causes compared to patients without CKD [1, 2, 3]. By the time patients with CKD develop end-stage renal disease and are in need of dialysis, many will have 1) evidence of coronary artery calcification (CAC), 2) structural cardiac abnormalities, and 3) abnormal cardiac diastolic function [4, 5, 6, 7, 8, 9, 10, 11]. While this can be explained, in part, by concomitant traditional CV risk factors, such as hypertension, diabetes mellitus, and hyperlipidemia, it is increasingly recognized that loss of kidney function independently promotes CV disease and potentially causes these changes in the heart and coronary vessels [12, 13, 14, 15]. CAC is associated with increased all-cause mortality in the general population, but, despite the high prevalence of coronary artery disease (CAD) in CKD-5D, a clear connection linking CAC to cardiac outcomes in CKD has not been established [16, 17, 18, 19, 20, 21]. 

We hypothesize that CAC impacts mortality in this patient population by adversely affecting global cardiac function. To this end, our objectives for this retrospective study are 1) to define the spectrum of cardiac structural and functional changes occurring in patients with CKD-5D and 2) to determine how strongly CAC is associated with the structural and functional cardiac manifestations in CKD-5D patients. 

## Subjects and methods 

Patients with CKD-5D were recruited from 37 dialysis centers across the state of Kentucky to participate in a large prospective cohort study evaluating the relationship between CAC and mineral bone disorder. Inclusion criteria were: age 18 years or older; chronic maintenance dialysis of at least 3 months duration; and calcidiol levels within normal range. Exclusion criteria included pregnancy; systemic illnesses or organ diseases (except diabetes mellitus); clinical conditions that may limit study participation (e.g., respiratory distress and infections); chronic alcoholism and/or drug addiction; participation in a study of an investigational drug during the past 90 days; on active transplant list; treatment within the last 6 months with drugs that may affect bone metabolism (except for treatment with calcitriol, vitamin D analogs, and/or calcimimetics). 

Out of this population, a parallel sub-study was designed as a prospective cohort followed to investigate the link between CAC and structural and functional changes within the heart. Over a 2-year period from 2012 to 2014, a total of 157 patients consented and were enrolled in this sub-study. Patients were required to undergo both echocardiography and noncontrast computed tomography (CT) scanning to evaluate cardiac morphology and function as well as CAC, respectively. The investigators adhered to the Declaration of Helsinki in the conduct of the study and registered the IRB-approved study with ClinicalTrials.gov (#NCT00859612). 

Blood samples were obtained from all participants, and the following traditional and nontraditional bone markers were measured: plasma intact parathyroid hormone (PTH), bone-specific alkaline phosphatase (BSAP), tartrate-resistant acid phosphatase-5b (TRAP-5b), sclerostin, Dickkopf-1 (DKK-1), and fibroblast growth factor-23 (FGF-23). Serum calcium and phosphorus levels were also measured. Methods employed to measure levels of these markers are as described previously [22]. 

CAC Agatston scores were obtained by noncontrast multiple-slice computed tomography using a SOMATOM 64 × 0.6 collimation scanner for all scans (Siemens, Washington, DC, USA). Images were acquired from the carina to the left ventricular (LV) apex. Scan parameters additionally included ECG gating, 120 kVp, 80 mAs/rotation, 0.33 s gantry rotation time, and 3 mm slice thickness. Images were analyzed on a 3D workstation using calcium-scoring software (Heart View CT, Siemens AG, Erlangen, Germany). Calcifications were identified as a plaque of ≥ 1 mm^2^ with a density of ≥ 130 HU and quantified by the Agatston scoring method [23]. Additionally, volumetric scores were obtained by multiplying the pixel area by the section thickness and were expressed as the sum of all lesion volumes in mm^3^. A square root of the volume transformation was performed to facilitate data interpretation (CAC SRVT (square root volume transformed)) [24]. Intraobserver error for interpretation of images was < 1% as determined through repeated interpretation 2 – 4 weeks apart. Interscan variability was < 3% through repeat measurements 30 minutes apart. 

Echocardiograms were performed by an experienced sonographer using a Phillips IE-33 ultrasonography machine (Phillips Medical Systems, Andover, MA, USA), with the patient breathing quietly in the left decubitus position. Resting blood pressure, heart rate, and morphometric measurements were obtained at the time of echocardiographic examination. Echocardiographic measurements were performed from standard American Society of Echocardiography (ASE) views, including the parasternal long axis and short axis, apical 4-chamber, apical 2-chamber, and sub-costal views. Two-dimensional images, flow and tissue Doppler images, and M-mode were analyzed. Tissue Doppler imaging was accomplished using a 2 mm-length sampling volume with the transducer positioned at the mitral annulus in both the lateral and septal positions from the apical 4-chamber view and anterior and inferior positions from the apical 2-chamber view. All reported cardiac morphological and functional measurements were obtained based on current ASE guidelines [25]. 

**Figure 1.**
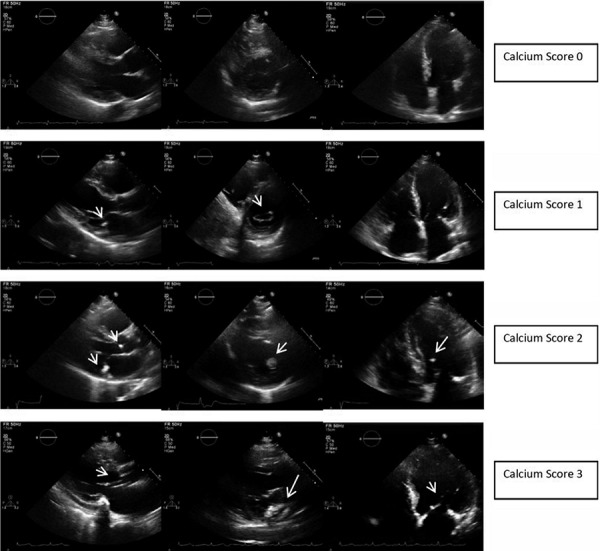
Echocardiographic cardiac valvular calcium scoring profile. Echocardiographic snapshots demonstrating the extent and severity of cardiac calcification, which was used to derive an overall calcification score. Cardiac calcification predominantly affected the mitral valvular annulus, leaflets, and chordae tendineae as well as the aortic valve leaflets and annulus.

Cardiac valvular calcification was defined as the presence of bright echogenic areas of at least 1 mm in length in the affected valve involving one or more leaflets of the mitral or aortic valves, mitral or aortic annulus, or the papillary muscles or chordal structures. The severity of calcification was graded on a semi-quantitative scale of 0 – 3, where 0 represents the absence of calcification, 1 represents mild calcification, 2 represents moderate calcification, and 3 represents severe calcification. Severity was judged by the number of leaflets involved (≤ 1 vs. > 1), the number of valves involved (≤ 1 vs. > 1), and the extent of involvement (punctate vs. diffuse calcification) (Figure 1). 

LV systolic function was determined by estimation of LV ejection fraction (LVEF) using the modified Simpson’s biplane method as detailed in the ASE guidelines [25]. In addition, the average peak systolic velocity measured by tissue Doppler imaging was recorded. Only measures in which tissue Doppler velocities were obtainable from the septal, lateral, anterior, and inferior aspects of the mitral annulus were used. Finally, global longitudinal strain of the LV was assessed from standard gray-scale images of the apical chamber views using speckle-tracking analysis software offline (Phillips IE-33, Phillips Medical Systems, Andover, MA, USA). 

LV diastolic function was assessed using mitral valve inflow Doppler as well as tissue Doppler imaging. The ratio of early passive LV diastolic filling (E) to late LV diastolic filling (A) was determined for initial classification of diastolic grade. The average of the septal and lateral mitral annular velocities from tissue Doppler imaging was used to determine the velocity of diastolic LV excursion corresponding to early LV diastolic filling (E’). This allowed for a calculation of the ratio of E:E’, which correlates with LV filling pressure. 

Statistical calculations were performed using SPSS™ software (IBM™ Corp., Armonk, NY, USA) version 22. Association between variables was calculated using Spearman’s Rho nonparametric test for correlation. Group comparisons of continuous variables were performed with nonparametric Mann-Whitney U or Kruskal-Wallis tests as appropriate. Dichotomous variables were compared using Fisher’s exact or χ^2^-tests, as appropriate. Due to the number of comparisons made, the significance threshold was set at p < 0.01. 

## Results 

A total of 157 CKD-5D patients were enrolled, for whom demographic and clinical information is shown in Table 1. Approximately 26% of participants reported a history of CAD, and 79% were treated for hypertension. Diabetics comprised approximately half of the study population. A total of 57% of the subjects reported a sedentary lifestyle, and over half of the population was obese (body mass index > 30 kg/m^2^). 

A total of 51% of patients had CAC scores greater than 400, 18% had CAC scores between 100 and 400, and 31% had CAC scores < 100, including 17% who had a CAC score equal to 0 (Table 2). Male subjects were found to have higher CAC scores than female subjects (median (interquartile range): 572 (110 – 2,292) vs. 209 (2 – 1,114), p = 0.009). CAC was strongly associated with age and reported history of CAD (rho = 0.447 and 0.432, respectively) by univariate analysis. Diabetics also had higher CAC scores (715 (range, 178 – 2,605) vs. 196 (range, 4 – 1,172); rho = 0.264, p < 0.01). The relationships between CAC and CAD, gender, and diabetes remained significant after adjustment for age. 

Among the structural parameters assessed using echocardiography, only cardiac valvular calcification was found to be strongly correlated with CAC by univariate analysis (rho = 0.405, p < 0.001) (Table 3). Valvular calcification was mostly localized to the mitral annulus, aortic annulus, and to lesser extents the mitral and aortic valve leaflets. Approximately 39% of the cohort had moderate or severe valvular calcification on the basis of our semiquantitative scoring system (score ≥ 2). Similar to CAC, cardiac valvular calcification score was also strongly correlated with age (rho = 0.358, p < 0.001), but, in contrast to CAC, it was not associated with diabetes status or male gender. Interestingly, no association was found between CAC or valvular calcification severity and LV size or left atrial volume. 

### Systolic LV function 

**Figure 2.**
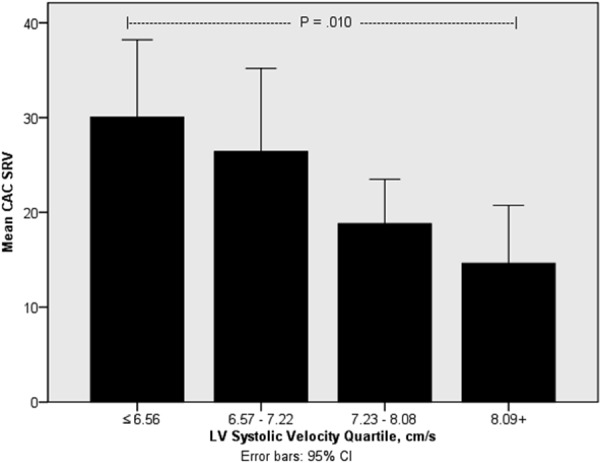
Mean coronary artery calcification (CAC) square root of the volume by left ventricular (LV) systolic velocity quartile. CAC scores were normalized as the square root of the volume and expressed according to peak LV systolic velocity quartiles expressed as cm/s using tissue Doppler imaging. CAC severity is associated with a progressive decrease in the peak LV systolic velocity obtained by tissue Doppler imaging reaching statistical significance between the lowest and highest quartile groups. These findings suggest the development of subclinical LV systolic dysfunction with CAC severity.

LVEF and peak LV systolic velocity were largely preserved in this cohort, however, within the range of peak LV systolic velocity values, they varied inversely with both CAC (rho = –0.259, p = 0.004) and cardiac valvular calcification score (rho = –0.218, p < 0.05) by univariate analysis (Figure 2) (Table 4). LV systolic velocity was not appreciably influenced by age, race, gender, or dialysis vintage but was associated with reported history of CAD (rho = –0.345, p < 0.001). Multivariable analysis demonstrated a significant negative relationship between CAC SRVT and peak LV systolic velocity after adjustment for age, diabetes status, gender, CAD status, and FGF-23 level (Std. B versus log-transformed LV systolic velocity = –0.321, p = 0.01). This relationship was modestly strengthened when only patients with LVEF > 40% were considered (Std. B = –0.343, p = 0.006), indicating that LVEF does not impact the association between CAC and peak LV systolic velocity. 

Approximately half of the study subjects were found to have abnormal values for global longitudinal strain (GLS). Similar to the findings for LV systolic velocity, GLS correlated with CAC (rho = 0.270, p = 0.003). As with LV systolic velocity, no relationship was found between GLS and age, race, or duration of CKD-5D. Notably, LV systolic velocity and GLS were more abnormal in study subjects with a history of CAD (mean 6.7 vs. 7.6, p < 0.001 and –13.9 vs. –16.8, p = 0.001, respectively). Multivariate analysis demonstrated CAC SRVT to be an independent predictor of GLS after adjustment for age and gender (Std. B 0.284, p = 0.014). Further adjustment for known CAD resulted in CAC no longer being predictive of GLS (B = 0.135, p = 0.209), likely due to their strong correlation. Taken together, these results suggest a link between CAC severity and LV systolic dysfunction. 

### Diastolic LV function 

**Figure 3.**
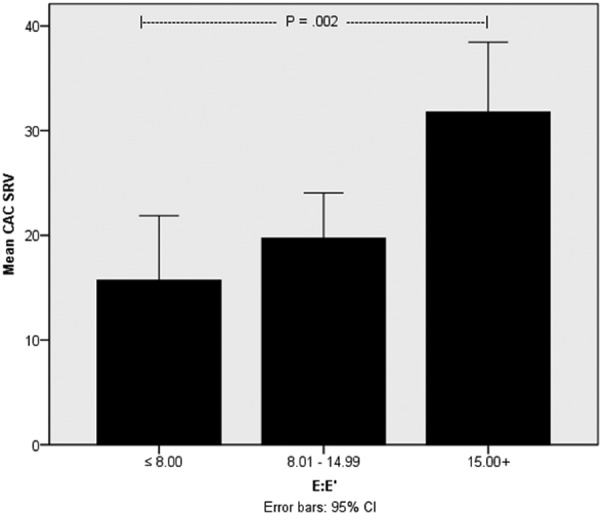
Correlation between coronary artery calcification (CAC) severity and left ventricular (LV) diastolic dysfunction. CAC severity was normalized as the square root of the volume to demonstrate a relationship with LV diastolic dysfunction as measured by the E:E’ ratio. E:E’ ratio values less than 8 are considered normal, whereas values ≥ 15 are considered abnormal and associated with abnormally-elevated LV filling pressure. Values falling between these two extremes are generally non-classifiable in the absence of other measures of LV diastolic function. Results show a strong association between CAC severity and abnormal LV diastolic function. When patients with significant mitral annular calcification were excluded from analysis, these findings remained significant.

In general, echocardiographic-derived measures of LV diastolic function indicate that more than 25% of this cohort have elevated LV filling pressure as defined by an E:E’ ratio ≥ 15. No relationship was found between age, race, gender, dialysis vintage, or history of CAD and E:E’. CAC and cardiac valvular calcification scores, however, demonstrate significant correlations by univariate analysis with estimates of LV filling pressure, a measure of LV diastolic function (E:E’, rho = 0.286, p = 0.001 and rho = 0.417, p < 0.001, respectively) (Figure 3). In addition to the association with CAC and cardiac valvular calcification, LV filling pressure estimates demonstrated correlation with LV mass index (rho = 0.448, p < 0.001), mitral valve inflow pattern (E:A ratio; rho = 0.354, p < 0.01), left atrial volume index (rho = 0.474, p < 0.001), LV end diastolic volume index (rho = 0.278, p = 0.001), systolic blood pressure (rho = 0.312, p < 0.001), and inferior vena cava dimension (rho = 0.282, p = 0.001) by univariate analysis. Adjusting for age, diabetes status, gender, and known CAD, CAC SRVT remained a significant predictor of LV diastolic dysfunction as measured by E:E’. Std. B = 0.317, p = 0.009) and LV systolic velocity (Std. B = –0.321, p = 0.01). Additional echocardiographic findings are summarized in Table 4. 

### Serum biochemical parameters 

We found no association between CAC and sclerostin, DKK-1, FGF-23, PTH, BSAP, and TRAP-5B, nor between these markers and cardiac function or structure. Interestingly, serum levels of FGF-23 correlated positively with GLS (rho = 0.335, p = 0.001) and negatively with LVEF (rho = –0.275, p = 0.003). FGF23 remained an independent predictor of GLS (Std. B = 0.235, p = 0.015) with adjustment for male gender, CAD, and dialysis vintage (n = 87). Multivariable analysis taking into account FGF-23 status did not alter the relationship between CAC SRVT and GLS or LV systolic velocity. No other biomarkers demonstrated associations with LV function. 

**Figure 4.**
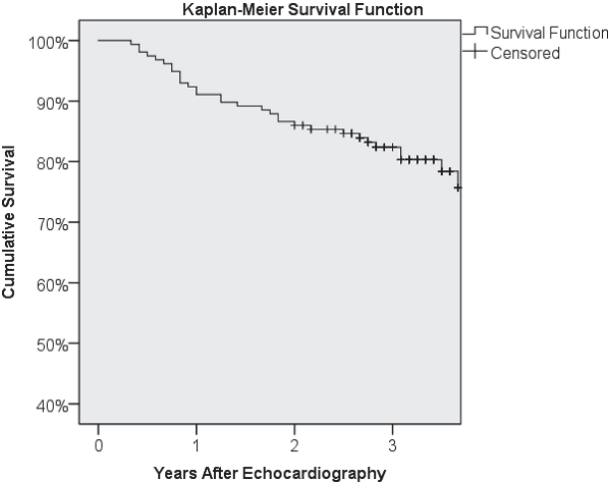
Kaplan-Meier survival. Overall survival shown by Kaplan-Meier survival curve as a function of time.

There were 27 deaths recorded among our cohort over the course of this study, representing a 17% mortality rate. The median duration of follow-up was 37 months (range, 4 – 76 months). The overall Kaplan-Meier survival curve is shown in Figure 4. One-year survival was 92% (157 patients exposed to risk), and 3-year survival was 87% (145 patients exposed to risk). Age, gender, race, dialysis vintage, and diabetes were not associated with survival in this study. History of CAD was a strong predictor of survival (3-year survival with CAD was 62 vs. 89% without). Patients with baseline CAC Agatston scores greater than 400 had a 1-year survival of 88 compared to 97% in patients with lower CAC scores, and a 3-year survival of 77 vs. 89%. However, by the 4^th^ year, the differences in survival narrowed to 74 vs. 79%, and the overall difference between the groups did not reach significance (Wilcoxon p = 0.067). Several echocardiography measures were independently predictive of survival, including advanced LV diastolic dysfunction associated with an elevated LV filling pressure (E:E’), peak LV systolic velocity, and GLS. LVEF did not impact survival in this population. 

**Figure 5.**
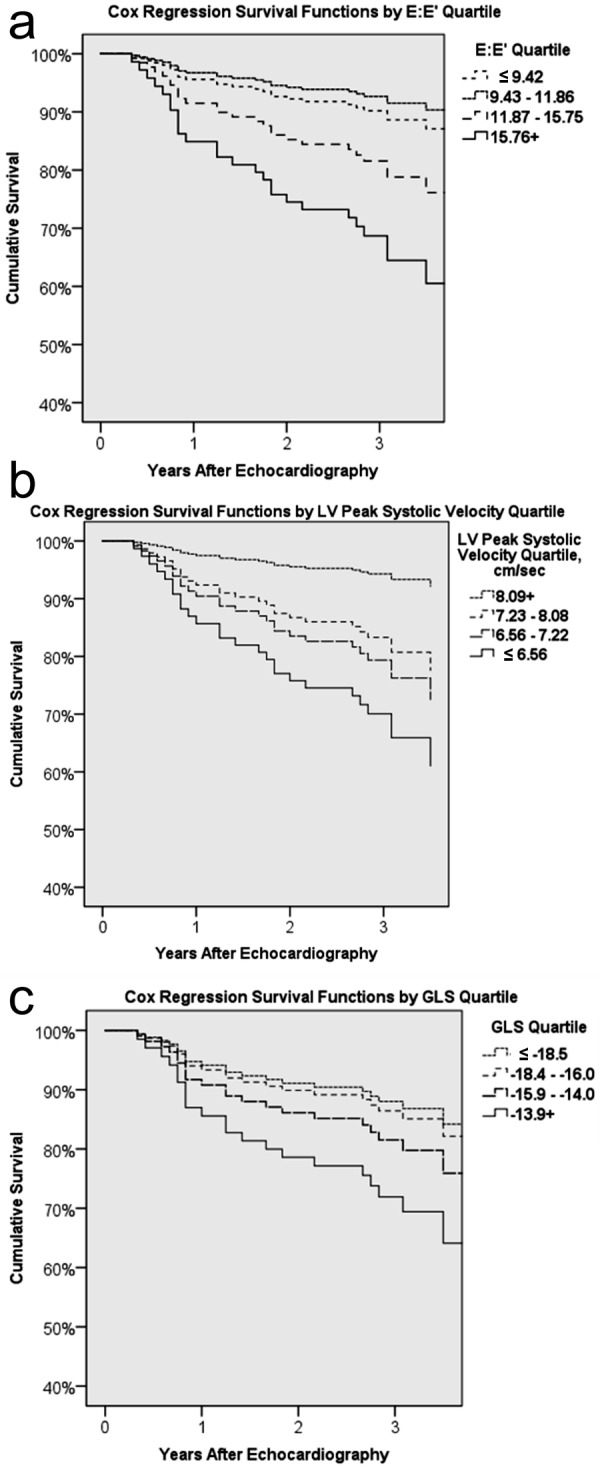
Figure 5a. Survival stratified by E:E’ quartiles. Cox regression survival functions stratified on the basis of E:E’ quartiles. E:E’ is an estimate of LV filling pressure and an indicator of the presence of advanced LV diastolic dysfunction. Values of E:E’ < 8 are considered normal whereas values equal to, or exceeding 15, are abnormal and consistent with elevated LV filling pressure. E:E’ is a strong predictor of survival in CKD-5D. Figure 5b. Cox regression survival functions stratified on the basis of peak LV systolic velocity quartiles. Peak LV systolic velocity is obtained using tissue Doppler echocardiographic imaging. Although the range of peak LV systolic velocity values is generally considered normal in this patient cohort, a significant relationship was seen between this measure and CAC burden and survival. Figure 5c. Survival functions by global longitudinal strain quartile from Cox regression analysis. Survival stratified by global longitudinal strain. Cox regression survival functions stratified on the basis of values of global longitudinal strain obtained from our CKD-5D patient cohort. GLS measures LV deformation during systole and is a sensitive marker of LV systolic function. More negative values are associated with normal LV systolic function. In our study, GLS was significantly associated with CAC burden and survival.

E:E’ remained a strong predictor of mortality after adjusting for CAD, LVEF, age, and diabetes status (E:E’ Z-score hazard ratio (HR) of 1.8; 95% CI 1.2 – 2.6; p = 0.003; N = 142). Figure 5a shows survival functions for each E:E’ quartile from Cox regression. The presence of significant valvular calcification precludes an accurate assessment of LV diastolic function. Therefore, a subanalysis in patients with no or mild valvular calcification only was performed, which resulted in an unappreciable increase in the relationship between E:E’ and survival (E:E’ Z-score HR 2.1 vs. 1.8 in overall population). 

Peak LV systolic velocity conferred a protective effect on mortality in this population (Figure 5b). After adjustment for CAD, LVEF, and age, the hazard ratio for survival associated with the Z-score of peak LV systolic velocity was 0.6 (0.4 – 0.9, p = 0.043; N = 128). Patients in the highest quartile of peak LV systolic velocity values had lower mortality (> 8.09 cm/s; 6.5%) compared with those in the lowest quartile of peak LV systolic velocity values (< 6.56 cm/s; 34.4%). GLS was also strongly associated with survival. More positive GLS values conferred increased mortality risk (HR 2.3; 95% CI 1.2 – 4.6; p = 0.013; N = 114) after similar adjustment for CAD, LVEF, and age. Patients in the highest quartile of GLS values had the highest mortality (> –13.9; 29.6%) compared to those in the lowest quartile (≤ –18.5; 15.2%) (Figure 5c). 

## Discussion 

In this cross-sectional, prospective study of contemporary CKD-5D patients, several key findings are evident: 1) CKD-5D patients as a group are faced with a significant CAC burden, not simply in terms of prevalence but also in relation to CAC severity, such that 69% of patients had CAC scores above 100, which has been associated with high risk of cardiovascular events, and over half were at very high risk, with CAC scores above 400; 2) detection of CAC is often linked with evidence of adverse cardiac systolic and diastolic function; 3) cardiac valvular calcification detected by echocardiography is common in CKD-5D is also tied to adverse cardiac function and may serve as a marker of CAC; and 4) CAC and adverse cardiac function are strong predictors of mortality in CKD-5D. 

Our cohort consisted of patients with advanced kidney disease on hemodialysis who, not surprisingly, also had other identifiable CV risk factors. Interestingly, while 26% of these patients reported a history of CAD, 83% were found to have evidence of CAC, and more than half had a CAC score above 400, which has been associated with an elevated risk of future CV events including myocardial infarction and death. 

Furthermore, our results demonstrate a significant association between CAC burden and LV function. Specifically, both LV systolic and diastolic function are adversely affected by the presence of CAC. Despite the fact that patients in our cohort were mostly found to have preserved LV systolic function on the basis of LVEF assessment alone, more sensitive measures of LV systolic function, including tissue Doppler-derived peak LV systolic velocity and speckle-tracking-derived GLS, demonstrate worsening LV systolic function as a function of CAC severity. Multivariate analysis adjusted for age, diabetes, gender, and CAD showed CAC to be a significant predictor of LV peak systolic velocity. Similarly, multivariate analysis demonstrated a significant and independent association between CAC and GLS after adjustment for gender and age. This relationship between CAC and cardiac function is further strengthened by an association between CAC burden and echocardiographic-derived measures of LV diastolic dysfunction. In fact, CAC burden was a significant predictor of advanced LV diastolic dysfunction as measured by LV filling pressure estimates (E:E’) after adjustment for age, diabetes status, and CAD. Taken together, our findings show the development of subclinical LV systolic and diastolic dysfunction in CKD-5D patients and that this is tied to underlying CAC, thereby corroborating and extending previous reports linking loss of kidney function with CAC and abnormal cardiac function [26, 27, 28, 29, 30, 31, 32, 33, 34, 35, 36]. 

In our cohort, the presence of advanced LV diastolic dysfunction (E:E’), low peak LV systolic velocity, or abnormal GLS was associated with worse survival. The strongest predictor of survival in our cohort was LV diastolic dysfunction. These factors remained significant predictors of survival even after adjustment for age, gender, CAD, and diabetes status. This is in contrast to a more recently-published study in patients with CKD-5 who were just starting hemodialysis [37]. In that study, LV diastolic dysfunction was also prevalent but was not a significant predictor of survival after multivariate analysis. Instead, right ventricular (RV) dysfunction was the strongest predictor of survival. Since we focused on the relationship between CAC and LV morphology and function, we did not perform a separate analysis of RV function. Our study is further distinguished from that study in that our study included patients with a longer dialysis vintage and involved more sensitive measures of LV systolic function, allowing us to be able to identify subclinical LV systolic dysfunction. 

Valvular calcification is more common in patients with CKD compared to persons without CKD, and so it is not surprising that a large proportion of the participants in our study have valvular calcification [38, 39]. Importantly, we found an association between cardiac valvular calcification and CAC consistent with previously-published data [19, 40, 41, 42, 43, 44, 45]. The strong association between valvular and vascular calcification suggests common molecular pathways and potentially-overlapping therapeutic targets. Furthermore, our findings show that the presence of significant cardiac valvular calcification as detected by echocardiography is of itself associated with subclinical LV systolic dysfunction and may explain the association between cardiac valvular calcification and mortality reported in earlier studies [19, 43, 44]. The significance of these findings for CKD patients is two-fold: First, detection of valvular calcification by echocardiography may serve as a marker for CAC, indicating a more severe coronary atherosclerotic burden. Secondly, like CAC, valvular calcification signals the onset of LV systolic dysfunction before it may be clinically apparent. Thus, routine echocardiography in CKD-5D patients provides important clinical information that potentially improves cardiovascular risk stratification. 

Finally, we assessed the association of echocardiographic parameters with serum levels of several bone markers known to be involved in the pathophysiology of chronic kidney disease – mineral and bone disorder (CKD-MBD). Among these, only FGF-23 was found to have a modest, but statistically – significant relationship with LV GLS after adjustment for age, gender, and CAD. The significance of this finding is not clear, although it is novel and potentially highlights the impact of the mineral bone disorder aspect of CKD on cardiac function. Other studies have shown a strong correlation between FGF-23 levels and left ventricular hypertrophy (LVH) in patients with CKD [46, 47, 48]. We were unable to find such an association in our cohort of CKD-5D patients. One explanation of this may be the high prevalence of LVH and the much higher serum levels of FGF-23 observed in our study participants as compared with those of other studies. Further studies are warranted to determine more precisely the mechanism tying these pathophysiological processes together and whether serum biomarkers of CKD-mineral and bone disorder, such as FGF-23, can be used clinically for better CV risk stratification. 

Some limitations of our study should be noted. First, this is a cross-sectional cohort of patients with CKD-5D who were clinically stable. Therefore, our findings may not be applicable to hospitalized patients, patients with milder stages of CKD, or post-kidney-transplant patients with CKD. These findings, however, are derived from a population representative of CKD-5D patients in a real-world outpatient setting with a significant proportion of African American and diabetic participants. In this regard, we believe that these findings will have relevance across the general CKD-5D population. Second, considering that a large proportion of the study population had evidence of mitral annular calcification, our ability to adequately assess LV diastolic function is limited. However, when patients with moderate to severe mitral annular calcification were removed from the analysis, the relationship between CAC, cardiac valvular calcification, and LV diastolic function remained. Thirdly, we report on 157 study participants among whom there were 27 deaths.  Despite the low absolute number of deaths, we were able to show a significant difference in survival on the basis of CAC score at years 1 and 3, but not at year 4. Since these events are spread over the span of 4 years, the number of patients available for analysis becomes smaller over time and may impair our ability to detect a statistically meaningful difference between the groups with higher vs. lower CAC scores.  Additionally, these patients have numerous other comorbidities that undoubtedly influence their risk of mortality, thereby potentially diluting the effect of CAC on survivability over time. 

In conclusion, CKD-5D is characterized by a high CAC burden, which is tied to adverse diastolic and systolic cardiac function and mortality. The findings underscore the importance of CAC and its serious clinical relevance in this patient population. Cardiac valvular calcification detected by echocardiography is common in these patients and is independently linked to both CAC severity and abnormal LV function. Finally, the bone biomarker FGF-23 is associated with subclinical LV systolic dysfunction. These findings support the value of a comprehensive echocardiographic evaluation for cardiovascular risk stratification in CKD-5D patients. 

## Conflict of interest 

The authors have no conflicts of interest to disclose. 

## Acknowledgments 

Research reported in this publication was supported by the National Institute of Diabetes and Digestive and Kidney Diseases of the National Institutes of Health under award number R01DK080770 and the Kentucky Nephrology Research Trust. The project was supported by the National Center for Research Resources and the National Center for Advancing Translational Sciences, National Institutes of Health, through Grant UL1TR000117. The content is solely the responsibility of the authors and does not necessarily represent the official views of the National Institutes of Health. 

We would like to acknowledge the contribution of the University of Kentucky echocardiography staff and most notably Paul A. League who served as principal sonographer on this study. We also recognize the research coordinators Kimberly McLaughlin, PA-C, and Nedda Hughes, PA-C, who enrolled the patients, recorded all data, and guided patients through all study activities. 

**Table 2.** Multiple-slice computed tomography and laboratory findings.

Variable	Mean	Range
CAC Agatston score	1,206	0.0 – 7,826
CAC square root of volume	23.7	0.0 – 77.4
Hemoglobin (g/dL)	11.1	8.4 – 16.1
High-density lipoprotein (mg/dL)	44	21 – 88
Low-density lipoprotein (mg/dL)	82	21 – 166
Fibroblast growth factor-23 (RU/mL)	8,000	227 – 28,000
Total parathyroid hormone (pg/mL)	342	8 – 1657
Tartrate-resistant acid phosphatase-5b, unit/L	6.8	0.7 – 16.7
Bone-specific alkaline phosphatase, unit/L	50.9	9 – 248
Dickkopf-1, pmol/L	35.8	0 – 147
Procollagen type 1 N-terminal propeptide, ng/mL	18.1	2.5 – 101.6
Sclerostin, pg/mL	175	20 – 709

CAC = coronary artery calcification.

**Table 1.** Baseline demographic and clinical characteristics.

Characteristics	Value (n = 157)
Number of patients	157
Age in years, median (range)	55 (20 – 84)
Months on dialysis, median (range)	45 (3 – 292)
Sex Male Female	93 (59%) 64 (41%)
Race/ethnicity Black White Other	83 (53%) 72 (46%) 2 (1%)
Body mass index, median kg/m^2^ (range)	32 (18 – 62)
Reported exercise ≥ 1/week	68 (43%)
Diabetes mellitus	75 (48%)
Hypertension	124 (79%)
Known coronary artery disease	40 (26%)
Current smoker	35 (22%)
Statins	58 (37%)
Cinacalcet	46 (29%)
Phosphate binders with calcium	66 (42%)
Active vitamin D	88 (56%)

**Table 3.** Correlation between cardiac valvular calcification and coronary artery calcification.

Rho correlation	CAC score	LV systolic velocity	GLS	E:E’
CAC score	–	–0.259^a^	0.270^a^	0.286^a^
Valvular calcification score	0.405^b^	–0.250^a^	0.085	0.417^b^

CAC = coronary artery calcification; LV = left ventricular; GLS = global longitudinal strain; E:E’ = ratio of passive LV filling peak flow velocity to average peak velocity of the septal and lateral mitral annulus by tissue Doppler imaging. ^a^p < 0.01 and ^b^p < 0.001.

**Table 4.** Echocardiographic measurements.

Parameter	Median	Range
Left ventricular ejection fraction (%)	61	9 – 83
LV systolic velocity (cm/s)	7.22	4.18 – 11.6
Global longitudinal strain	–16.0	–24 – –5.5
Left ventricular mass index (g/m^2^)	128	49 – 227
Relative wall thickness ratio (%)	53.9	24.7 – 119
LVEDVI (mL/m^2^)	57.1	23.2 – 140
Left atrial volume index (mL/m^2^)	39.5	15.4 – 111
E:A	1.05	0.57 – 4.07
E:E’	11.86	5.64 – 43.48
Inferior vena cava dimension (cm)	2.0	1.0 – 3.4
Cardiac index (liters/min/m^2^)	3.04	1.02 – 6.73
Stroke volume index (mL/m^2^)	40.5	13.8 – 83.0

All measurements were made using two-dimensional, pulsed- and continuous-wave Doppler, and color and tissue Doppler methods according to the American Society of Echocardiography standards. LV = left ventricular; LVEDVI = left ventricular end diastolic volume index; E:A = ratio of early passive left ventricular peak flow velocity to late filling peak velocity due to atrial contraction during diastole; E:E’ = ratio of the early passive left ventricular filling peak flow velocity to the tissue-Doppler-derived average peak systolic velocity during the early passive filling phase in diastole.
